# Supplementary material for: The impact of history of depression and access to weapons on suicide risk assessment: a comparison of ChatGPT-3.5 and ChatGPT-4
Source: PeerJ. 2024 May 29;12:e17468. doi: 10.7717/peerj.17468 (PMC11143969; doi:10.7717/peerj.17468)
Supplement: Supplemental Information 3 — Examples of interactions with ChatGpt-3.5 and ChatGPT-4 with conditions and languages, showing the input and output inserted into the models. [file peerj-12-17468-s003.docx]

Attached are examples of interactions with ChatGpt-3.5 and ChatGPT-4 with conditions and languages, showing the input and output inserted into the models.

ChatGpt-3.5:

<https://chat.openai.com/share/6938095f-f6dd-4ee5-bd58-4d608f523040>

ChatGPT-4:

<https://chat.openai.com/share/257a49a6-177b-42ed-9e17-3e0e7ba94877>
